# Time‐lapse monitoring of mouse embryos produced by injecting sonicated, frozen‐thawed sperm heads with high or low chromosomal integrity

**DOI:** 10.1002/rmb2.12319

**Published:** 2020-02-18

**Authors:** Yoshihisa Harada, Masayuki Kinutani, Toshitaka Horiuchi

**Affiliations:** ^1^ Graduate School of Comprehensive Scientific Research Prefectural University of Hiroshima Hiroshima Japan; ^2^ Kinutani Women’s Clinic Hiroshima Japan

**Keywords:** intracytoplasmic sperm injection, mouse, sonication, sperm chromosome, time‐lapse imaging

## Abstract

**Purpose:**

To investigate the first‐division kinetics and in vitro development of embryos produced by injecting sonicated sperm heads with high or low chromosomal integrity into oocytes.

**Methods:**

Mouse spermatozoa were frozen after separating the sperm heads from the tails by sonication in an EGTA solution (EGTA group) or M2 medium (M2 group). The chromosomal integrity of sonicated mouse spermatozoa was analyzed by injecting the sperm heads into fresh mouse oocytes. The developmental potential of spermatozoa was examined by injecting the sperm heads into vitrified‐warming mouse oocytes. We used a time‐lapse monitoring system to compare the first‐division kinetics.

**Results:**

Chromosomal integrity was preserved significantly more frequently in the EGTA group (90.6%) than in the M2 group (32.7%). Blastocysts developed significantly more often in the EGTA group (80.8%) than in the M2 group (39.6%). In the M2 group, with frequent chromosome aberrations, the time between the sperm injection and first cleavage was delayed (18.4 hours), compared to the EGTA group (16.5 hours). All results of the EGTA group were similar to that of fresh epididymal spermatozoa.

**Conclusion:**

The EGTA solution for sonication maintained the integrity of sperm chromosomes. Our results revealed a relationship between sperm chromosome integrity and first‐division kinetics.

## 1 INTRODUCTION

Intracytoplasmic sperm injection (ICSI) has been widely used, both in reproductive engineering in laboratory animals and for assisted reproductive applications in humans.^1^ In 1976, for the first time, Uehara and Yanagimachi reported that injecting human sperm or hamster sperm into hamster oocytes could form a pronucleus.^2^ In mice, Kimura and Yanagimachi succeeded in obtaining offspring by performing ICSI with a piezo‐micromanipulator.^3^

In humans, the first pregnancy achieved with ICSI was reported in 1992.^1^ Currently, clinical ICSI applications are used for treating male infertility and IVF failure. In addition, it is possible that ICSI might be used to fertilize oocytes with high probability of success, even with DNA‐damaged sperm. Several studies have shown that damage to sperm DNA was associated with fertilization, embryo development, embryo quality, miscarriage after in vitro fertilization (IVF), and ICSI.^4^, ^5^, ^6^, ^7^, ^8^, ^9^ Sperm selection is an important problem in modern embryology. However, there is no established method for detecting sperm chromosome damage non‐invasively in order to select undamaged sperm for IVF.

With an application of a time‐lapse system, embryonic development can be monitored at all times without removing embryos from the incubator. This system enables continuous observation of either embryo morphokinetics or division speed, and thus, these parameters can be measured.^10^, ^11^ With a mouse spermatozoon that have a clear sperm chromosome status, it is possible to use the time‐lapse system to investigate the effect of sperm chromosome status on embryo development and division speed. This approach could lead to resolution of human male infertility problems.

Sonication of mouse spermatozoa can readily separate sperm tails and heads without using a piezo pulse.^12^ However, sonication may increase the frequency of sperm chromosome aberrations. As a matter of course, mouse spermatozoa are highly sensitive to damage. It is thought that the Ca^2+^‐dependent DNase is activated by time course after sonication, and activated DNase causes damage to sperm chromosomes.^13^ In addition, chromosomal stability in mouse spermatozoa is affected by the sonication medium. It has been reported that suspending mouse spermatozoa in a simple Tris‐HCl–buffered solution that contains 50 mmol/L ethylene glycol‐bis (‐aminoethyl ether) ‐ N, N, N, N ‐ tetraacetic acid (EGTA) and 50 mmol/L NaCl (Tris‐HCl EGTA) could maintain chromosome integrity during freeze‐drying or freezing without cryoprotection.^14^, ^15^ In the golden hamster, sperm chromosome integrity was maintained after sonicating frozen‐thawed spermatozoa in Tris‐HCl EGTA.^16^ However, it is not clear whether this solution might reduce chromosomal abnormalities in mouse sperm heads that are separated from spermatozoa by sonication.

The purpose of present study was to investigate the effect of Tris‐HCl buffer with EGTA on the integrity of mouse sperm chromosomes after sonication. Furthermore, we examined the effect of sperm chromosome damage on developmental competence and morphokinetic parameters in mouse oocytes fertilized by ICSI with sonicated frozen‐thawed sperm heads.

## 2 MATERIALS AND METHODS

### 2.1 Preparation of mouse oocytes

In this study, we used B6D2F1 female mice, 8‐12 weeks of age. Superovulation was induced by intraperitoneal injection of 5 IU pregnant mare serum gonadotropin (Serotropin; Asuka Pharmaceuticals). At 48 hours after serotropin injection, the female mice received an injection of 5 IU human chorionic gonadotoropin (hCG; Asuka Pharmaceuticals). Cumulus‐oocyte complexes (COCs) were collected from oviducts between 15 and 16 hours after the hCG injection. The collected COCs were immediately denuded by pipetting with 250 IU/mL hyaluronidase. We assessed denuded oocyte maturity, based on the presence of the first polar body. The MII oocytes were cultured in an incubator at 37.0°C under 6% CO_2,_ 5% O_2_ and 89% N_2_ for approximately 30 minutes, until vitrification. These experiments were approved by the Committee for Ethics on Animal Experiments at the Prefectural University of Hiroshima, Japan (18A010).

### 2.2 Oocyte vitrification and warming

Oocytes were vitrified according to the method described by Kuwayama et al^17^ Briefly, MII oocytes were placed in equilibration solution containing 7.5% (v/v) ethylene glycol (EG, Sigma‐Aldrich), 7.5% (v/v) dimethyl sulfoxide (DMSO, Nacalai Tesque Inc.) in mHTF (Irvine Scientific), supplemented with 20% (v/v) SSS (Serum Substitute Supplement: Irvine Scientific) for 15 minutes at 25‐28°C. After equilibration, MII oocytes were transferred to a vitrification solution containing 15% (v/v) EG, 15% (v/v) DMSO, and 0.5 mol/L sucrose (Sigma‐Aldrich) in mHTF supplemented with 20% (v/v) SSS for 1 minute at 25‐28°C. Ten oocytes were loaded onto cryotops (Kitazato Supply) at a minimum volume and then immediately dipped into liquid nitrogen for storage.

The oocyte warming procedure was performed by immersing the tip of the cryotop directly into a 1.0 mol/L sucrose solution for 1 minute at 37°C. Next, the oocytes were transferred to a 0.5 mol/L sucrose solution for 3 minutes and then a 0.25 mol/L sucrose solution for 5 minutes. Next, oocytes were washed once in mHTF supplemented with SSS for 5 minutes at 37°C. Prior to the ICSI, warmed oocytes were cultured for approximately 60 minutes in 50 μL global total medium (LifeGlobal) at 37°C under 6% CO_2_, 5% O_2_, and 89% N_2_.

### 2.3 Sperm collection and sperm freezing after sonication

For sperm freezing and sonication, we used M2 medium^18^ or an EGTA solution, which contained 10 mmol/L Tris‐HCl buffer with 50 mmol/L ethylene glycol‐bis (‐aminoethyl ether) ‐ N, N, N, N ‐ tetraacetic acid (EGTA).^14^, ^15^

Spermatozoa were collected from the cauda epididymis of B6D2F1 mice, 12‐16 weeks of age. Control spermatozoa were suspended in approximately 1 mL of global total/HEPES medium (LifeGlobal). Spermatozoa were allowed to swim up into this medium for up to 5 minutes at 37°C under 5% CO_2_ in the air before collection of upper 100 μL of the medium. On the other hand, experimental spermatozoa were suspended in approximately 1 mL of M2 medium, supplemented with 1 mg/mL bovine serum albumin (BSA), or an EGTA solution, for up to 5 minutes at 37.0°C under 5% CO_2_ in air. Spermatozoa were centrifuged at 500 × *g* for 5 minutes, the supernatant was removed, and spermatozoa were mixed with 1 mL of M2 medium or EGTA solution. This suspension was transferred to ice water (0°C), then sonicated for 5 seconds at 50% sonicator output (20 kHz, VP‐5S; Taitec).

More than 90% of mouse spermatozoa underwent head and tail separation. We placed 100‐μL aliquots of sonicated spermatozoa, in M2 medium (M2 group) or EGTA solution (EGTA group), into 0.25 mL cryostraws, and froze them in liquid nitrogen (−196°C). Immediately before the piezo‐ICSI procedure, the frozen sperm heads were thawed at room temperature (25‐28°C).

### 2.4 Piezo‐ICSI procedure

We used an inverted microscope (ECLIPSE Ti‐U, Nikon), equipped with a micromanipulator (Narishige Inc.) and a Piezo impact drive (MB‐U, Prime tech Ltd.). The Piezo drive unit was driven by a controller (PMAS‐ET150, Prime Tech Ltd.). Approximately 7 μL fluorinert (FC‐770, 3M) was injected with a flat tip pipette (PINU07‐20FT, Prime tech Ltd.) attached to an air injector (IM‐11‐2, Narishige Inc.). The inner diameter of the pipette was 5.95 μm, and the outer diameter was 7 μm. Frozen‐thawed sperm heads were suspended in 7% polyvinylpyrrolidone (PVP: Irvine Scientific) at a 1:1 ratio before injection. We transferred 10 oocytes into 5 μL droplets of mHTF that contained 10% SSS, which had been placed next to sperm head‐containing droplets (10 μL) covered with mineral oil (Irvine Scientific). The zona pellucida was penetrated with a piezo pulse (speed 2, intensity 2); then, to puncture the oocyte plasma membrane, we used one piezo pulse (speed 1, intensity 1). The Piezo‐ICSI procedure was completed within 1 hour of sperm thawing. Sperm head‐injected oocytes were incubated in 50 μL droplets of global total, under mineral oil, at 37.0°C in 6% CO_2_, 5% O_2_, and 89% N_2_ for 96 hours.

### 2.5 Preparation and analysis of chromosomes in frozen‐thawed sonicated sperm heads

We analyzed sperm chromosomes by injecting the sonicated and frozen‐thawed sperm heads into fresh mouse oocytes with the Piezo‐ICSI procedure, as described by Morishita et al^16^ Between 5 and 6 hours after sperm head injection, eggs with 2 polar bodies and 2 pronuclei (2PB + 2PN) were transferred into the global total medium, supplemented with 0.01 μg/mL vinblastine (Sigma‐Aldrich) to prevent male and female pronuclear fusion. At 19 and 21 hours after the Piezo‐ICSI, the oocytes that had arrested at first cleavage metaphase were treated with 1% pronase (Kaken Pharmaceutical CO., LTD.) for 7 minutes at room temperature (25‐28°C) to remove the zonae pellucidae. They were then treated with a hypo‐osmotic solution (1:1 mixture of 1% sodium citrate and 30% (v/v) fetal bovine serum) for 7‐8 minutes at 37°C. Chromosomes were spread out on clean slides, according to the gradual fixation/air‐drying method.^19^ The chromosome slides were stained with 2% (v/v) Giemsa solution (Merck) in buffered saline solution (pH 6.8) for 8 minutes.

### 2.6 Time‐lapse imaging and annotations

Embryos were individually cultured in an incubator equipped with a time‐lapse recording system (PrimoVision, Vitrolife) for approximately 4‐5 days, from the ICSI procedure up to the blastocyst stage. Each embryo was cultured in a 100 μL drop of global total in a culture dish with 16 microwells. Images of the embryos were recorded automatically every 10 minutes. Monopronucleated zygotes, multipronucleated zygotes, unfertilized, and degenerated oocytes were excluded from the measurements. Data were collected for the following time points: the time to second polar body appearance (2PB); the time to male and female pronuclei appearance (PNa); the time to male and female pronuclei disappearance (PNd); and the time to first division (2‐cell).

### 2.7 Statistical analysis

Statistical analyses were performed with GraphPad PRISM 6.03 software (GraphPad Inc.). For binary variables, Fisher's exact probability test and the chi‐square test were used to determine statistical differences. For continuous variables, we used the one‐way ANOVA and Tukey‐Kramer. Significant differences were assumed to be present at *P* < .05.

## 3 RESULTS

### 3.1 Chromosomal analysis of sonicated, frozen‐thawed sperm heads

**Figure 1:**
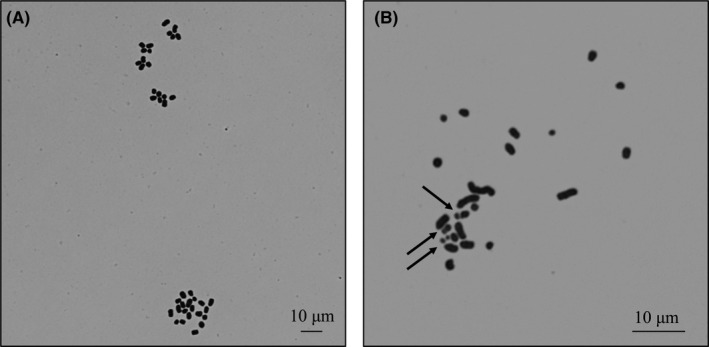
Chromosomes of frozen‐thawed mouse spermatozoa after sonication in either EGTA solution or M2 medium, evaluated after injecting into mouse oocytes. A, The normal number of 40 chromosomes, from sperm and oocyte pronuclei. B, Abnormal chromosomes are likely to originate from damaged sperm. Arrows point to chromosome fragments, derived from chromosome breaks

A total of 40 chromosomes per oocyte were judged to be normal (Figure 1A). The proportion of normal (unaltered) chromosomes was significantly (*P* < .01) higher in the EGTA group (90.6%) than in the M2 group (32.7%; Table 1). The chromosome normality of the EGTA group was similar to that of fresh epididymal spermatozoa (90.6% vs 97.4%, NS). Thus, the integrity of sperm chromosomes was maintained in the EGTA solution during sonication. Most chromosome aberrations were structural abnormalities (Figure 1B).

**Table 1:** Chromosomal analysis of mouse spermatozoa that were sonicated and frozen‐thawed in M2 medium or EGTA solution and then injected into mouse oocytes

Medium used for sonication and storage	No. of zygotes analyzed	No. (%) of oocytes with a normal karyotype	No. of oocytes with chromosome aberrations*	
			Structural	Aneuploidy
Control (Fresh)	39	38 (97.4)^a^	1	0
M2	49	16 (32.7)^b^	32	1
EGTA	53	48 (90.6)^a^	4	1

Note

In this experiment, we used mouse fresh oocytes.

Fresh epididymal spermatozoa use for ICSI before preservation served as controls.

EGTA: 100 mmol/L Tris‐HCl‐buffered solution + 50 mmol/L EGTA.

^a,b^Different superscript indicates significant differences (*P* < .01). Percentages of oocytes with normal sperm chromosomes were analyzed by Fisher's exact probability test and chi‐square test.

* Structural chromosome aberrations included chromosome/chromatid‐type breaks and exchanges.

### 3.2 Developmental competence of vitrified‐warmed mouse oocytes injected with sonicated mouse sperm heads

Table 2 summarizes the results of in vitro embryo development after injecting sonicated, frozen‐thawed sperm heads into mouse vitrified‐warmed oocytes. The proportions of oocytes that survived after ICSI, reached the 2‐cell stage, and reached the 4‐cell stage, were not significantly different between the M2 and EGTA groups. The proportions of embryos that developed from 2‐cell stage to the 8‐cell stage and blastocyst stage were significantly higher in the EGTA group than in the M2 group (62.3% vs 88.5% and 39.6% vs 80.8%, respectively). In the EGTA group, the proportions of embryos that developed to blastocyst stage were similar to that of fresh epididymal spermatozoa (80.8% vs 88.0% NS).

**Table 2:** In vitro development of mouse oocytes fertilized by intracytoplasmic injection of sperm heads that were sonicated and frozen‐thawed in M2 medium or EGTA solution

Medium used for sonication and storage	No. of oocytes injected with sperm	No. (%) of oocytes that survived after ICSI	2‐cell	No. (%) of embryos that developed†		
				4‐cell/2‐cell	8‐cell/2‐cell	Blastocyst/2‐cell
Control (Fresh)	52	50 (96.2)^a^	50 (100.0)^a^	48 (96.0)^a^	48 (96.0)^a^	44 (88.0)^a^
M2	60	57 (95.0)^a^	53 (93.0)^a^	43 (81.1)^b^	33 (62.3)^b^	21 (39.6)^b^
EGTA	60	56 (93.3)^a^	52 (92.9)^a^	46 (88.5)^ab^	46 (88.5)^a^	42 (80.8)^a^

Note

In this experiment, we used mouse vitrified‐warmed oocytes. EGTA: 100 mmol/L Tris‐HCl buffered solution + 50 mmol/L EGTA.

Fresh epididymal spermatozoa use for ICSI before preservation served as controls.

^a,b^Different superscripts indicate a significant difference (*P* < .05), based on Fisher's exact probability test and the chi‐square test; 2‐cell: first division.

^†^ Gives the numbers of embryos that developed to a from b, written as a/b; 4‐cell: second division; 8‐cell: third division.

### 3.3 Time to first division of vitrified‐warmed mouse oocytes injected with sonicated mouse sperm heads, detected with time‐lapse imaging

**Figure 2:**
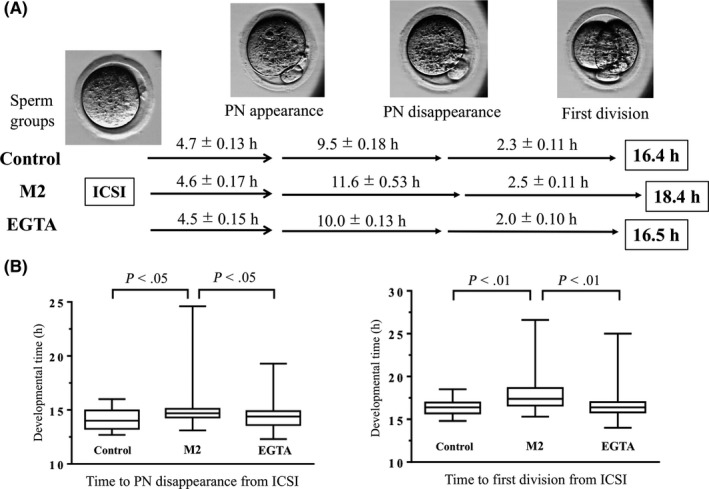
First‐division kinetics of embryos fertilized with an intracytoplasmic sperm injection (ICSI) of frozen‐thawed mouse spermatozoa, after sonication in M2 medium or an EGTA solution. A, Time‐lapse images show the different stages of embryo development (*top*) and the times to reach each stage (*bottom*). B, Comparison of the times to reach different developmental stages for the three groups. PN: pronuclei. *P* < .05, one‐way ANOVA and Tukey‐Kramer

Table 3 summarizes the results acquired from time‐lapse imaging up to the first division. No significant difference was found between groups for the 2PB and the PNa. However, the PNd was significantly delayed in the M2 group (15.8 hours) compared to the EGTA group (14.5 hours). The time to first division (2‐cell) was significantly delayed, by approximately 2 hours, in the M2 group (18.4 hours) compared to the EGTA group (16.5 hours,* P* < .01). Also, in the EGTA group, the first‐division kinetics of embryos were similar to that of fresh epididymal spermatozoa. The group with high chromosomal aberrations (M2 group) exhibited delays in the time from ICSI to PNd and the time from PNa to 2‐cell, compared to the EGTA group (Figure 2).

**Table 3:** First‐division kinetics of mouse oocytes fertilized by intracytoplasmic injection of sperm heads that were sonicated and frozen‐thawed in M2 medium or EGTA solution

Developmental stage	Mean [±SD] time to each stage (h)		
	Control (Fresh) n = 29	M2 n = 68	EGTA n = 55
2nd polar body (2PB)	1.8 [±0.05]^a^	1.8 [±0.10]^a^	1.9 [±0.08]^a^
Pronuclear appearance (PNa)	4.7 [±0.13]^a^	4.6 [±0.17]^a^	4.5 [±0.15]^a^
Pronuclear disappearance (PNd)	14.1 [±0.17]^a^	15.8 [±0.54]^b^	14.5 [±0.17]^a^
First division (2‐cell)	16.4 [±0.18]^a^	18.4 [±0.46]^b^	16.5 [±0.21]^a^
2PB to PNa	2.9 [±0.12]^a^	2.8 [±0.10]^a^	2.6 [±0.11]^a^
PNa to PNd	9.5 [±0.18]^a^	11.6 [±0.53]^b^	10.0 [±0.13]^a^
PNd to 2‐cell	2.3 [±0.11]^a^	2.5 [±0.11]^b^	2.0 [±0.10]^a^

Note

In this experiment, we used mouse vitrified‐warmed oocytes.

Fresh epididymal spermatozoa use for ICSI before preservation served as controls.

^a,b^Different superscript indicates not significant differences (*P* < .05). For comparison between three groups, one‐way ANOVA and Tukey‐Kramer were used.

## 4 DISCUSSION

This study showed that the injection of sperm heads with severe chromosomal damage into oocytes induced delays in the times to reach the PNd and 2‐cell stages. We measured the first‐division kinetics accurately with time‐lapse analysis and revealed a relationship between first‐division kinetics and sperm chromosome integrity. In mice, chromosomal abnormalities were previously reported to delay DNA replication, alter the cell cycle, and change the division time. 20, 21 The relationship between the recognition and repair of DNA damage in oocytes has been described previously.^22^ The results of this study indicated that sonicating spermatozoa in M2 medium caused chromosome fragmentation. Consequently, the time necessary to replicate DNA led to a high probability that the first cleavage time would be delayed. Indeed, the in vitro development of mouse oocytes fertilized by sperm heads was significantly lower in the M2 group than in the EGTA group. Moreover, the M2 group had remarkably lower percentages of 8‐cell stage and blastocysts compared to the EGTA group. It is likely that the damaged sperm DNA was not fully repaired during the first division. Arresting cleavage at 4‐cell stage may be related to delayed first cleavage. The chromosomal fragmentation observed in this study was a relatively large abnormality that could be observed by analyzing chromosomes with a light microscope. These results showed that the integrity of sperm chromosomes strongly influenced embryo development. Indeed, the in vitro development rates were low for embryos produced with damaged sperm chromosomes. The rates of blastocyst development per 2‐cell embryo in the M2 and EGTA groups (Table 2) appeared to be similar to the percentages of intact sperm chromosomes in each group (Table 1). However, in this study, we did not check the integrity of embryonic chromosomes in the cleavage and blastocyst stages. Although not shown in the results, the developmental time to reach the blastocyst stage after ICSI was longer in the M2 group (88.9 hours) than in the EGTA group (84.0 hours, *P* < .01). However, not all chromosomal abnormalities in spermatozoa after sonication in M2 medium can be observed under a microscope. Thus, undetected abnormalities could have affected the developmental rates. The euploidy of blastocysts should be studied in more detail in future investigations.

In humans, time‐lapse analyses showed that the timing of the first division influenced embryonic development.^23^, ^24^, ^25^, ^26^, ^27^, ^28^, ^29^, ^30^, ^31^ The mechanism for adjusting the timing of the first division remains incompletely understood. It was shown that the human meiotic spindle characteristics were associated with the first cleavage rate.^32^ The relationship between the first‐division timing and sperm chromosome integrity in humans remains unclear, but it has been shown that high sperm DNA fragmentation had an effect on the timing of the first division in humans.^33^ Those results were consistent with the results from the present study, based on mouse spermatozoa. However, a method for evaluating spermatozoa without morphologically damaging DNA remains to be established. The exact state of the DNA in each single sperm used for ICSI remains unclear, but this can only be evaluated by analyzing division kinetics or embryo development. Therefore, the timing of the first division, recorded with time‐lapse imaging, might be used as an indicator for objective, noninvasive evaluations of embryo quality.

In the present study, we demonstrated that the sonication medium affected the integrity of sperm chromosomes. We evaluated the integrity of sperm chromosomes by injecting sonicated sperm heads into mouse oocytes. We showed that the EGTA solution could maintain the integrity of mouse sperm heads during sonication. In contrast, when spermatozoa were sonicated in M2 medium, we detected serious damage to mouse sperm chromosomes. We speculated that the difference in chromosomal damage might have depended on the activation of DNase after sonication and/or after thawing, rather than the physical trauma due to sonication or freeze‐thaw treatment. The presence of a Ca^2+^‐dependent endonuclease in mouse spermatozoa was previously described by Mione et al^34^ In the EGTA group, the EGTA would have chelated the Ca^2+^ in solution, and the absence of Ca^2+^ during thawing and sonication probably inhibited DNase activation in the sperm heads. This inhibition could have contributed to the improvement in chromosome stability. In contrast, the M2 medium contained 1.71 mmol/L Ca^2+^, and it was much less effective for maintaining sperm chromosome integrity during thawing and sonication.

We found that the EGTA solution could cryopreserve spermatozoa without the addition of a cryoprotection agent. The EGTA solution maintained the integrity of sperm chromosomes, even during sonication. The spermatozoa used for ICSI is typically collected from the minimum number of males possible, and a large number of spermatozoa can be stored in LN_2_ without cryopreservation agents. Sonication does not require a piezo pulse to separate the sperm heads and tails; therefore, ICSI can be completed in a very short time.

In conclusion, we demonstrated that the EGTA solution was useful for maintaining mouse sperm chromosome integrity during sonication. Compared to the EGTA group, the M2 group had a higher rate of chromosomal aberrations after spermatozoa sonication. Moreover, compared to the EGTA group, the M2 group exhibited significant delays to PNd and to the 2‐cell stage after ICSI. As a result, a low percentage of the embryos with severe sperm chromosomal damage developed into blastocysts. These results also indicated that time‐lapse monitoring was a useful tool in assessing the PNd and the time to the 2‐cell stage for predicting sperm chromosome integrity.

## DISCLOSURES

*Conflict of interest*: The authors declare no conflict of interest. *Human rights statements and informed consent*: This study did not include human participants. *Animal studies*: All the experiments were approved by the Committee for Ethics on Animal Experiments at the Prefectural University of Hiroshima, Japan (18A010).
